# Integrated analysis reveals the potential of cluster of differentiation 86 as a key biomarker in high-grade glioma

**DOI:** 10.18632/aging.205359

**Published:** 2023-12-26

**Authors:** Xuebin Wen, Chaochao Wang, Zhihao Pan, Yao Jin, Hongcai Wang, Jiang Zhou, Chengfeng Sun, Gengfan Ye, Maosong Chen

**Affiliations:** 1 Department of Anesthesiology, Ningbo Medical Center Lihuili Hospital, Ningbo 315100, Zhejiang, China; 2 Department of Radiology, Ningbo Medical Center Lihuili Hospital, Ningbo 315100, Zhejiang, China; 3 Department of Neurosurgery, Ningbo Medical Center Lihuili Hospital, Ningbo 315100, Zhejiang, China

**Keywords:** CD86, high-grade glioma, biomarker, prognosis, radiomics

## Abstract

This study aimed to evaluate the potential of cluster of differentiation 86 (CD86) as a biomarker in high-grade glioma (HGG). The TCGA and TCIA databases were used to obtain the CD86 expression value, clinical data, and MRI images of HGG patients. Prognostic values were assessed by the Kaplan-Meier method, Receiver operating characteristic curve (ROC), Cox regression, logistic regression, and nomogram analyses. CD86-associated pathways were also explored. We found that CD86 was significantly upregulated in HGG compared with the normal group. Survival analysis showed a significant association between CD86 high expression and shorter overall survival time. Its independent prognostic value was also confirmed. These results suggested the possibility of CD86 as a biomarker in HGG. We also innovatively established 2 radiomics models with Support Vector Machine (SVM) and Logistic regression (LR) algorithms to predict the CD86 expression. The 2 models containing 5 optimal features by SVM and LR methods showed similar favorable performance in predicting CD86 expression in the training set, and their performance were also confirmed in validation set. These results indicated the successful construction of a radiomics model for non-invasively predicting biomarker in HGG. Finally, pathway analysis indicated that CD86 might be involved in the natural killer cell-mediated cytotoxicity in HGG progression.

## INTRODUCTION

Glioma is one of the most common primary solid tumors in the central nervous system, which leads to a serious health burden in terms of morbidity and mortality. The high-grade glioma (HGG, WHO grade III and IV tumors) has a strong degree of differentiation, invasion, and migration [1], and it generally causes a poor prognosis of patients with a median survival for grade IV glioma of 16.0 months [2]. Especially, the multifocal HGG shows the most unfavorable prognosis. The current treatment strategies in clinical for HGG include radiotherapy, temozolomide chemotherapy, and gross total resection [3]. However, the best treatment approach has not been established and the clinical outcome of HGG patients achieved limited improvement [4]. In addition, the occurrence of chemo- and radiotherapy resistance has become a challenge for developing the progress of glioma treatments. Identification of the regulatory mechanism and formulation of effective treatments for HGG is imperative. It is urgent to explore useful molecular signatures to improve the survival of HGG patients.

The cluster of differentiation 86 (CD86), also called B7-2, encodes a type I membrane protein that is a member of the immunoglobulin superfamily. CD86 is expressed alone on human monocytes, and it is the ligand for two proteins at the cell surface of T cells, cytotoxic T-lymphocyte-associated protein 4 (CTLA4) and CD28 antigen [5]. The binding of CD86 with CD28 antigen can activate the T-cell, but CTLA4 can compete with CD28 for binding CD86 on antigen-presenting cells to limit T-cell activation and diminish the immune response [6]. However, CD86 has a different interaction with both receptors (CD86-CD28 ~20μM and CD86-CTLA-4 ~2μM) [7]. It follows that CD86-CTLA4 binding is closely related to immune suppression. Therefore, anti-CTLA4 immunotherapy, which impedes the CD86-CTLA4 binding, contributes to the release of the T cell activity inhibition, enhancement of the immune response, and improvement of the patient’s prognosis. A better understanding of the regulation of CD86 supports the development of biomarkers for response prediction to anti-CTLA4 immunotherapy [8]. CD86 itself has been regarded as a potential target for immunotherapy [9, 10]. Previous studies also suggested that CD86 may interfere with the immune system by activating and depleting immune functions, which might be the reason why CD86 positivity was related to the worse prognosis [11]. In addition, CD86 was an unfavorable prognostic biomarker in lower-grade glioma (LGG) [12] and glioblastoma (GBM) patients [13]. It follows that CD86 is an important biomarker commonly associated with prognosis and immunotherapy. Detection and prediction of CD86 has profound clinical value.

At present, few researchers reported the value of CD86 in HGG. In this study, we first evaluated the potential of CD86 as a molecular biomarker in HGG. The expression pattern and prognostic value of CD86 in HGG were assessed. We also explored the possible regulatory mechanism associated with CD86 involved in HGG progression. Further, this study innovatively developed a non-invasively prediction model on the CD86 expression based on the radiomics features of MRI images. This study aims to provide a promising biomarker for HGG and develops a non-invasive prediction method, which contributes to evaluating patients’ prognosis and immunotherapy response, as well as stratifying the risk populations with poor prognosis, guiding neoadjuvant immunotherapy, and improving their quality of life.

## MATERIALS AND METHODS

### Data source of HGG patients and expression analysis on CD86

This study first performed the pan-cancer analysis to explore the expression level of CD86 in tumor and normal tissues. Then the expression of CD86 in patients with HGG was explored. The HGG patients were selected from the GBM and LGG datasets in the Cancer Genome Atlas (TCGA) database (https://portal.gdc.cancer.gov/). A total of 1114 samples of TCGA_GBM and TCGA_LGG were obtained from the TCGA database, and the inclusion criteria of HGG patients were as follows: 1) had the data of tumor grade; 2) WHO grade III and grade IV; 3) had the data of survival time and survival status; 4) survival time≥1 month; 5) had the data of age, gender, IDH mutation status, Chr_1p_19q codeletion, MGMT methylation status, radiotherapy history and chemotherapy history; 6) primary tumor and had gene expression RNA-Seq data. Finally, 298 patients who had the above complete information were enrolled in our further analysis. The baseline data of 298 HGG patients was presented in Table 1.

**Table 1:** The baseline characteristics of HGG patients stratified with CD86 expression.

	**Subgroup**	**Total (N=298)**	**Low (n=150)**	**High (n=148)**	**P**
Age (year)	<60	197	108	89	0.041
	≥60	101	42	59	
Gender	female	120	61	59	0.982
	male	178	89	89	
Grade	III	170	108	62	<0.001
	IV	128	42	86	
IDH status	wildtype	169	61	108	<0.001
	mutant	129	89	40	
Chr_1p_19q	non-codel	248	102	146	<0.001
	codel	50	48	2	
MGMT promoter status	unmethylated	118	38	80	<0.001
	methylated	180	112	68	
Radiotherapy	no	79	37	42	0.552
	yes	219	113	106	
Chemotherapy	no	70	41	29	0.150
	yes	228	109	119	

Then the expression difference of CD86 in normal and HGG groups was analyzed. In addition, the expression pattern of CD86 in subgroup populations of patients with HGG was determined after stratifying patients by clinical characteristics, including different age (<60, ≥60), gender (male, female), IDH mutation status (wildtype, mutant), Chr_1p_19q codeletion (none-codel, codel), MGMT methylation status (unmethylated, methylated), radiotherapy history (yes, no) and chemotherapy history (yes, no). Further, all the HGG patients were divided into 2 groups according to CD86 expression level (high, low), and the association between CD86 expression and the above clinical characteristics was evaluated by Logistic regression analysis.

### Prognostic value of CD86

The prognostic value of CD86 in HGG was then explored. Firstly, all HGG patients were divided into CD86 high and low expression groups according to the best cut-off value of CD86 expression level by maxstat R package. Then the Kaplan-Meier analysis by R survival package and log-rank test were performed to explore the association of CD86 expression with the overall survival (OS) time of patients. The receiver operating characteristic curve (ROC) analysis by R package pROC was used to assess the prediction performance of CD86 on OS. In addition, we also verified the prognostic value of CD86 in HGG using the data from the Chinese Glioma Genome Atlas (CGGC) database (http://www.cgga.org.cn/).

To further disclose the prognostic value, we performed a subgroup survival analysis on CD86 regarding different clinical characteristics based on TCGA data. Cox regression analyses by survival R package were then used to explore the association of variables with OS and the independent prognostic value of CD86. We also transformed the CD86 expression value into the ordinal variable and explored the association between CD86 expression groups and OS with or without adjusting confounders by Logistic regression analysis.

### Comprehensive nomogram model establishment

Based on the results of multivariable Cox regression analysis, we also established a comprehensive nomogram model containing independent factors (including CD86) and obtained the risk score of each patient by the rms R package. The Kaplan-Meier analysis and log-rank test were used to explore the survival difference between high and low risk score groups. ROC and Decision Curve Analysis (DCA) analyses were performed to evaluate the performance of the nomogram model in HGG.

### Pathway enrichment analysis on CD86

Due to the importance of CD86 in HGG, this study subsequently explored the potential regulatory mechanism associated with CD86. The c2.cp.kegg.v7.4.symbols.gmt was obtained from the Molecular Signature Database (http://www.gsea-msigdb.org/gsea/msigdb/index.jsp). All patients were divided into high and low expression groups according to the best cut-off value of CD86 expression value, and then Gene set enrichment analysis (GSEA) was used to evaluate the significant pathway enriched in 2 groups. Further, a single sample Gene Set Enrichment Analysis (ssGSEA) algorithm was performed to quantify the significant pathway score of each sample in HGG. The correlation between CD86 and pathway score was explored.

### Immune infiltration analysis on CD86

This study also explored the correlation of CD86 expression with immune infiltrates in HGG. Through the gene expression profile, we obtained the immune score of 22 immune cells by the CIBERSORT algorithm. The Pearson method was used to assess the correlation of CD86 expression level with the abundance of immune cells. Further, the immune cells with significant correlation with CD86 were then selected for comparing the difference in infiltration level between CD86 high and low expression groups.

### The source of radiomics data

Due to the potential of CD86 as an important biomarker in HGG, we innovatively explored the possibility of radiomics characteristics of MRI images for predicting CD86 expression without invasiveness. A total of 458 patients with MRI images in TCGA_GBM and TCGA_LGG datasets were obtained from the Cancer Imaging Archive (TCIA) (https://public.cancerimagingarchive.net/nbia-search/) database. The inclusion criteria of images were as follows: 1) enhanced MRI CE-T1WI images; 2) preoperative images; 3) had sufficient quality; 4) had corresponding TCGA data. Finally, 89 HGG patients with complete TCGA data and MRI images were selected for further radiomics analysis.

### Region of interest segmentation and features extraction of MRI images

Two experienced radiologists, blinded to the clinical data of the patients, performed region of interest (ROI) segmentation using 3D Slicer software. Then the open-source Python package PyRadiomics was used to extract the radiomic features. The extracted features included (1) shape-based features, such as three-dimensional size and shape of the ROI; (2) first-order features, describing the distribution of the voxel intensities within the ROI using commonly used and basic metrics; and (3) texture features, including the gray-level co-occurrence matrix (GLCM), gray level dependence matrix (GLDM), gray-level run-length matrix (GLRLM), gray-level size-zone matrix (GLSZM), neighboring gray-tone difference matrix (NGTDM). In total, 107 radiomics features were extracted from each HGG image. The intraclass correlation efficient (ICC) was performed to evaluate the consistency of the (ROI) segmentation by two physicians. Finally, 84 radiomics features with ICC≥0.8 were selected for further analysis.

### Radiomics model establishment and performance validation

Before model establishment, 89 HGG patients with complete TCGA data and MRI images were assigned to the training set and validation set according to the 6:4 ratio, and their baseline distribution between the training set and validation set had no difference. Subsequently, a radiomics model was constructed in the training set. To determine the more important features among 84 radiomics features, we used the mRMR (Maximum relevance, minimum redundancy) algorithm by mRMRe R package to filter radiomics features among them. After mRMR analysis, 20 radiomics features were identified. Subsequently, we performed RFE (Recursive feature elimination) algorithm on 20 features by caret package to obtain the optimal feature sets. RFE analysis can rank all the radiomics features and gradually remove the less important factors. After RFE analysis, the optimal features were determined and used for the further prediction model establishment.

Further, we used the optimal features to construct the radiomics model for predicting CD86 expression in the training set. Support Vector Machine (SVM) and Logistic regression (LR) were the common algorithms to establish the machine learning model in recent studies [14–16]. Therefore, this study only used these 2 methods to develop the prediction model based on the radiomics features. SVM and LR algorithms were performed with caret and glm R packages, respectively, and the variables’ importance in the two models was assessed. Further, the radiomics score (RS) integrating the optimal features in 2 models was calculated. Calibration curve, ROC, precision-recall (PR) curve, and DCA were performed to evaluate the performance of 2 radiomics models in the training set. Further, ROC, PR, and DCA were also performed in the validation set to assess the stability of our established models. Further, the RS between CD86 high and low expression groups was also compared based on the whole population.

### Comprehensive model establishment containing radiomics score and clinical characteristics

This study further explored the clinical value of the radiomic model in HGG. Based on the RS from the SVM method, the Kaplan-Meier method and log-rank test were performed to explore the survival difference between RS high and low groups. The multivariable Cox regression analysis was conducted to identify the independent factors associated with the OS among RS and clinical characteristics. Then the significant independent factors were enrolled into the establishment of a comprehensive nomogram model. ROC and Calibration curves were performed to evaluate the performance of the comprehensive model for predicting the prognosis of HGG patients.

### Statistical analysis

The data used in this study were analyzed with SPSS software and R packages. The t-test and χ^2^ test were used to compare the quantitative and qualitative data between the 2 groups, respectively. The association of CD86 with clinical characteristics and death risk was analyzed with Logistic regression. The Pearson method was used to explore the correlation between CD86 expression and the infiltration level of immune cells. The survival difference of the Kaplan-Meier curve was compared with the log-rank test. Univariable Cox regression analysis was used to explore the correlation of variables with OS time, and multivariable Cox regression analysis was used to determine the independent factors. P value<0.05 was considered as a statistically significant difference.

### Availability of data and materials

The dataset used and/or analyzed during the current study is available from the corresponding author on reasonable request.

## RESULTS

### The expression of CD86

**Figure 1:**
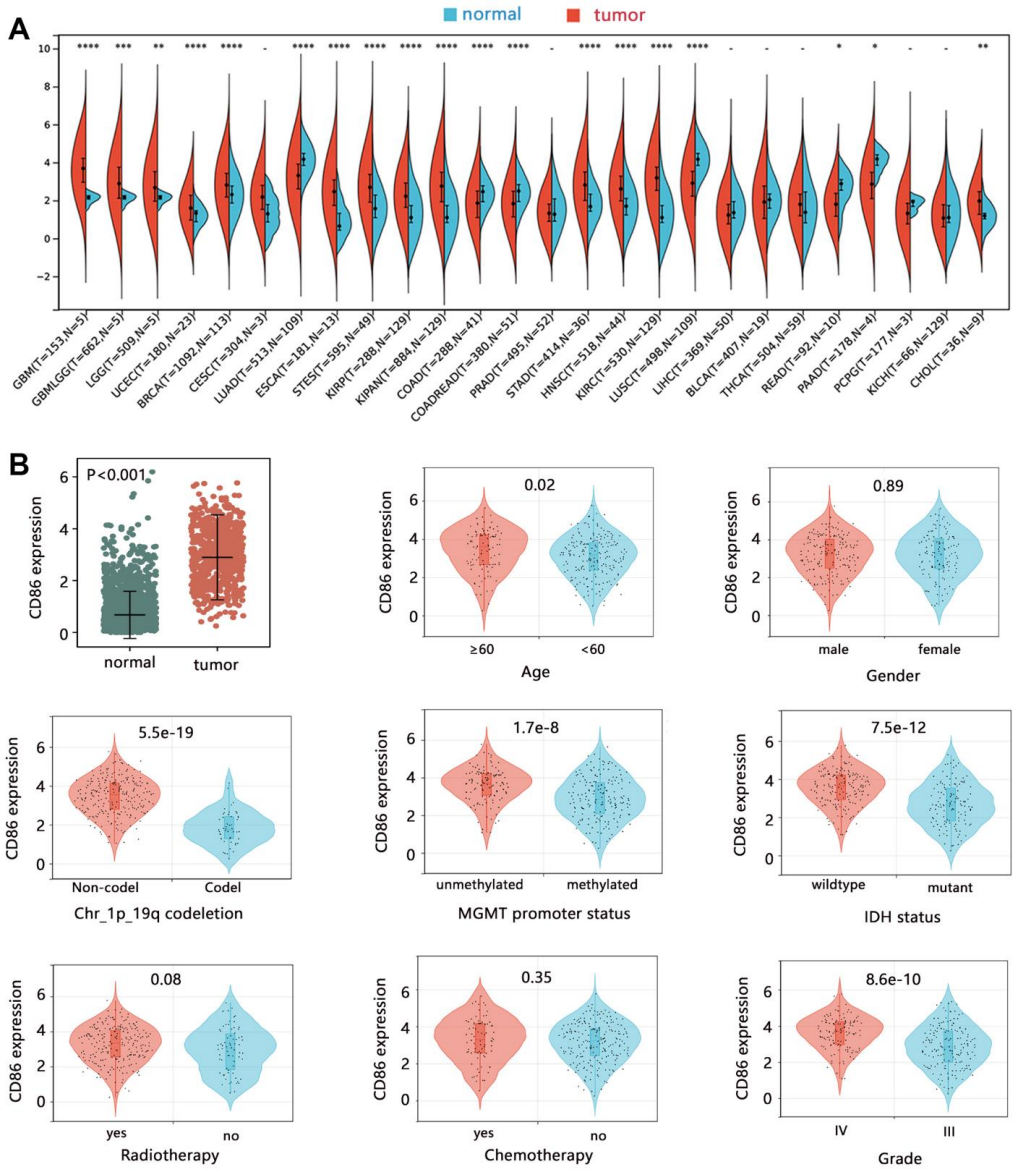
**The expression analysis of CD86 mRNA.** The expression difference of CD86 in (**A**) pan-cancer, and (**B**) HGG tissues.

This study first explored the expression of CD86 in human cancers. The pan-cancer analysis showed that CD86 was aberrantly expressed in most cancers compared with that in normal tissues (Figure 1A). Regarding HGG, CD86 was significantly upregulated in tumor tissues (Figure 1B, P<0.001). We also compared the expression difference of CD86 among HGG patients based on different clinical characteristics, finding that the expression of CD86 showed no difference in different genders, with or without radiotherapy and chemotherapy groups (all P>0.05). Higher expression was significantly observed in patients with age≥60 years, non-codel of Chr_1p_19q, unmethylated MGMT promoter, wildtype IDH, and grade IV (all P<0.05). Logistic regression analysis further indicated the correlation of CD86 with these characteristics (Table 2).

**Table 2:** The association of CD86 with clinical characteristics among HGG patients.

	**β**	**OR (95%CI)**	**P**
Age (≥60 vs <60)	0.585	1.795 (1.101, 2.926)	0.019
Gender (female vs male)	-0.105	0.900 (0.564, 1.436)	0.658
Grade (IV vs III)	1.440	4.222 (2.582, 6.906)	<0.001
IDH (mutant vs wildtype)	-1.442	0.236 (0.144, 0.389)	<0.001
Chr_1p_19q codeletion (codel vs non-codel)	-3.378	0.034 (0.008, 0.144)	<0.001
MGMT (methylated vs unmethylated)	-1.298	0.273 (0.167, 0.447)	<0.001
Radiotherapy (yes vs no)	0.526	1.692 (0.975, 2.936)	0.061
Chemotherapy (yes vs no)	-0.133	0.875 (0.522, 1.467)	0.613

Abbreviation: OR, odds ratio; CI, confidence interval.

### The prognostic value of CD86

**Figure 2:**
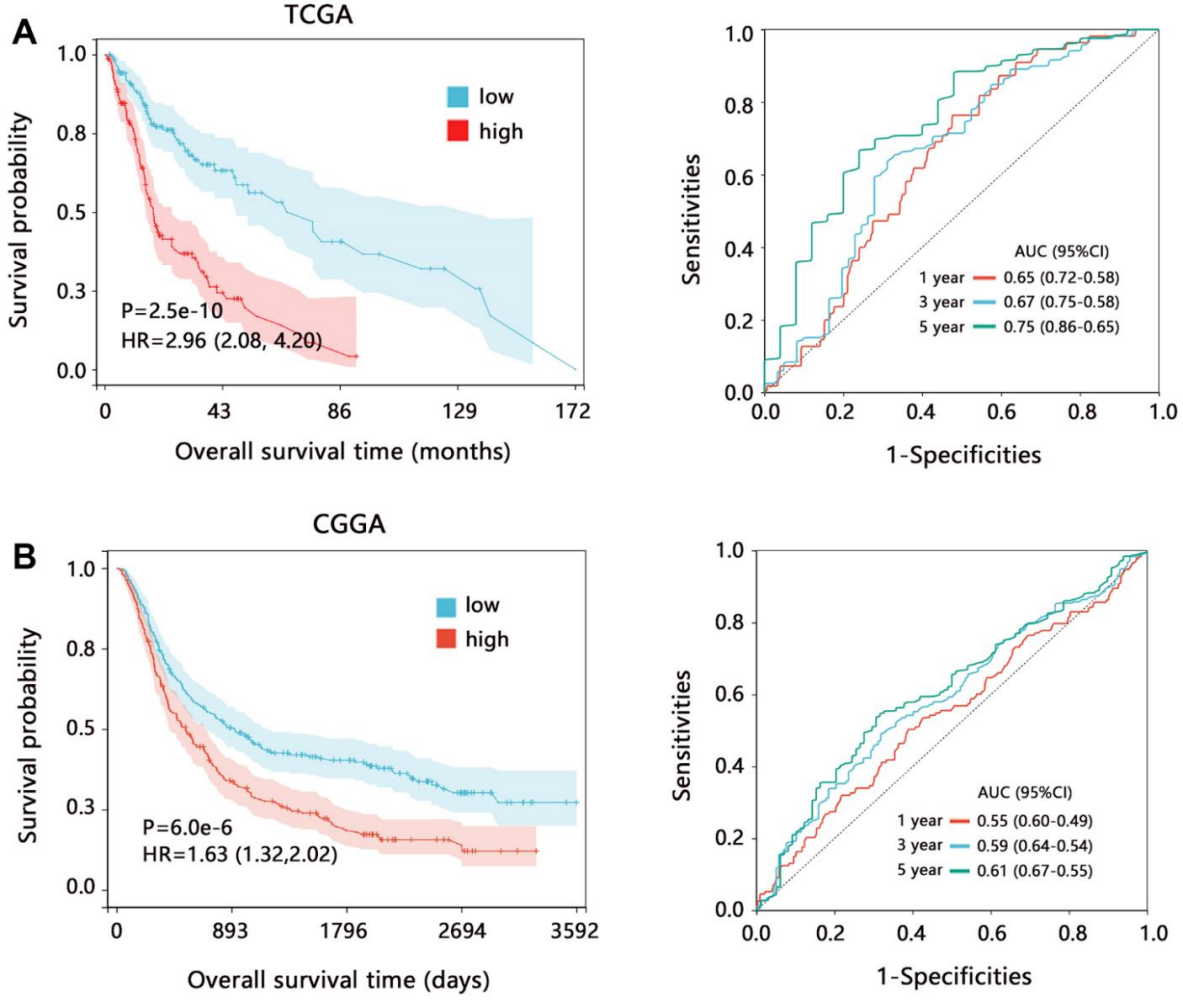
**The prognostic value of CD86 in HGG regarding the overall survival time.** Kaplan-Meier analysis and ROC analysis based on the data from (**A**) TCGA and (**B**) CGGA databases. Abbreviation: HR, Hazard ratio; CI, confidence interval; AUC, area under curve.

This study further assessed the potential clinical value of CD86 from the aspect of prognostic impact. The survival analysis showed that high expression of CD86 was significantly related to the poor OS time of HGG patients (Figure 2A, HR=2.96, P<0.001). The ROC analysis showed the prediction ability of CD86 on the 1, 3, and 5-year overall survival. We also used the data of CGGA to verify the clinical value of CD86 in HGG, and the results were consistent with that of TCGA (Figure 2B). These results confirmed the clinical value of CD86 in HGG.

**Figure 3:**
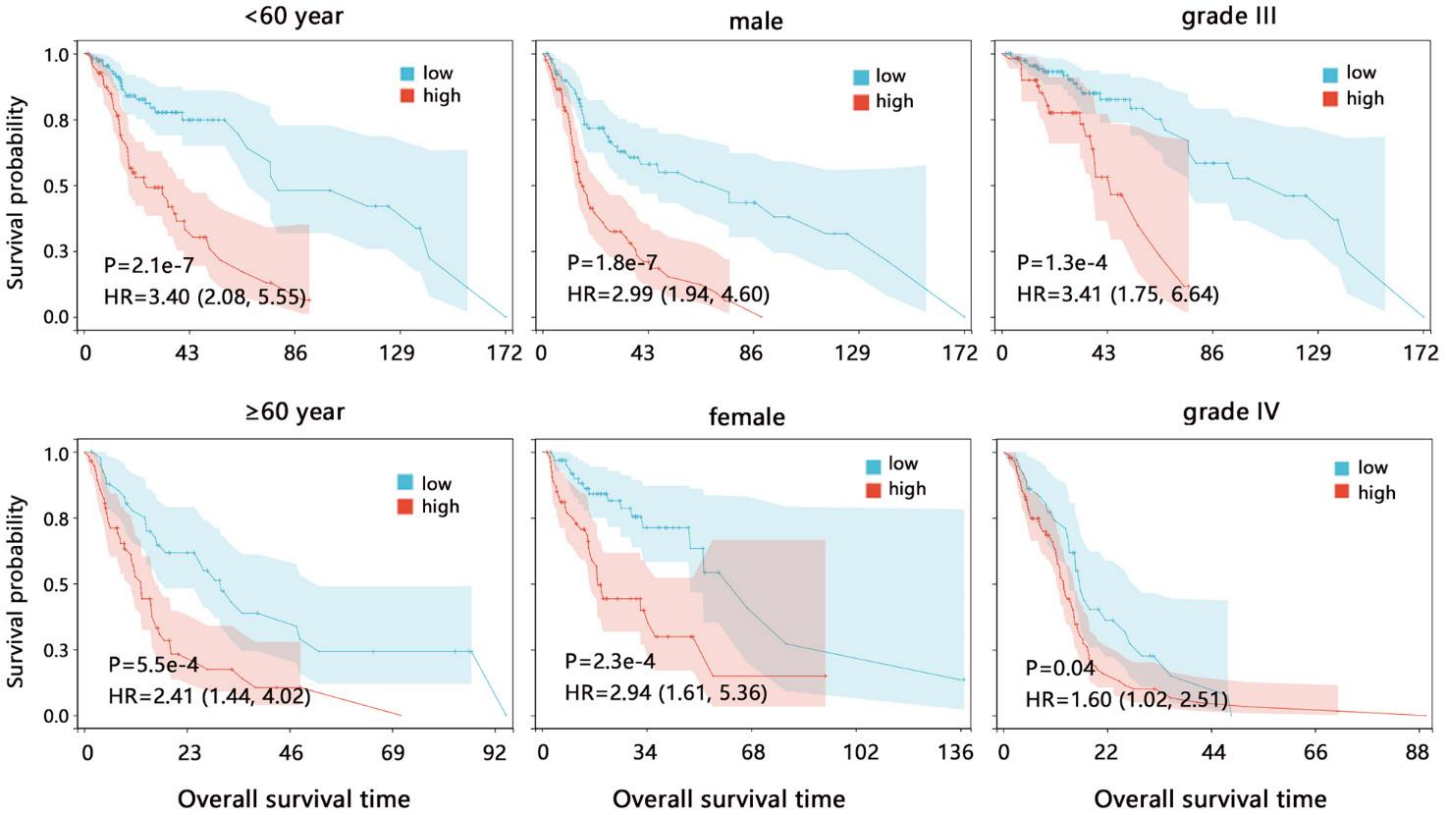
**The Kaplan-Meier survival analysis of CD86 in different subgroups among HGG patients.** Abbreviation: HR, Hazard ratio; CI, confidence interval.

We also assessed the prognostic value of CD86 on the overall survival in different subgroups based on TCGA data. Survival analysis further showed that CD86 high expression was significantly associated with poor prognosis in all subgroups (Figure 3, all HR>1, all P<0.05). These results indicated that CD86 can favorably distinguish the patient’s prognosis.

To further verify the prognostic value of CD86, we performed a Cox regression analysis. The univariate Cox regression analysis (Table 3) showed that 7 variables including CD86 (HR=1.604, P<0.001) were significantly related to the OS of HGG patients. We then enrolled the variables with P<0.05 into the multivariable Cox regression analysis, finding that CD86 was independently related to the prognosis of HGG patients (HR=1.213, P=0.035). We then translated the CD86 expression value into a qualitative variable and explored the correlation of the CD86 group with patients’ death risk, finding that the death risk of HGG patients in the crude model increased with the increase of CD86 expression (Table 4, P for trend<0.001). After adjusting the confounders of age and gender, the correlation was still observed (P for trend=0.002). Especially when the expression of CD86 was larger than 3.745, HGG patients had twice as death risk as these patients with CD86 expression less than 2.678 (OR=2.657, P=0.002).

**Table 3:** Cox regression analysis about overall survival on CD86 and clinical characteristics.

	**Univariate**		**Multivariate**	
	**HR (95%)**	**P**	**HR (95%)**	**P**
CD86	1.604 (1.368, 1.880)	<0.001	1.213 (1.014, 1.451)	0.035
Age (≥60 vs <60)	2.633 (1.887, 3.674)	<0.001	1.162 (0.799, 1.689)	0.433
Gender (female vs male)	0.807 (0.569, 1.144)	0.228		
Grade (IV vs III)	9.012 (6.085, 13.348)	<0.001	6.360 (4.033, 10.030)	<0.001
IDH (mutant vs wildtype)	0.174 (0.115, 0.265)	<0.001	0.496 (0.285, 0.862)	0.013
Chr_1p_19q (non-codel vs codel)	0.185 (0.093, 0.369)	<0.001	0.681 (0.286, 1.622)	0.386
MGMT (methylated vs unmethylated)	0.355 (0.253, 0.497)	<0.001	1.109 (0.756, 1.628)	0.595
Radiotherapy (yes vs no)	1.232 (0.826, 1.838)	0.306		
Chemotherapy (yes vs no)	0.549 (0.389, 0.775)	0.001	0.421 (0.292, 0.608)	<0.001

Abbreviation: HR, Hazard ratio; CI, confidence interval.

**Table 4:** The correlation between CD86 and the death risk of patients.

**Group**	**Crude model**		**Adjusted model**	
	**OR (95%CI)**	**P**	**OR (95%CI)**	**P**
<2.678	reference		reference	
2.678-3.745	1.511 (0.855, 2.672)	0.155	1.535 (0.845, 2.790)	0.159
>3.745	2.886 (1.619, 5.146)	<0.001	2.657 (1.448, 4.876)	0.002
P for trend	1.699 (1.272, 2.268)	<0.001	1.629 (1.203, 2.207)	0.002

Adjusted model: with the adjustment of age and gender. Abbreviation: OR, odds ratio; CI, confidence interval.

### Comprehensive nomogram model establishment containing CD86 and clinical characteristics

**Figure 4:**
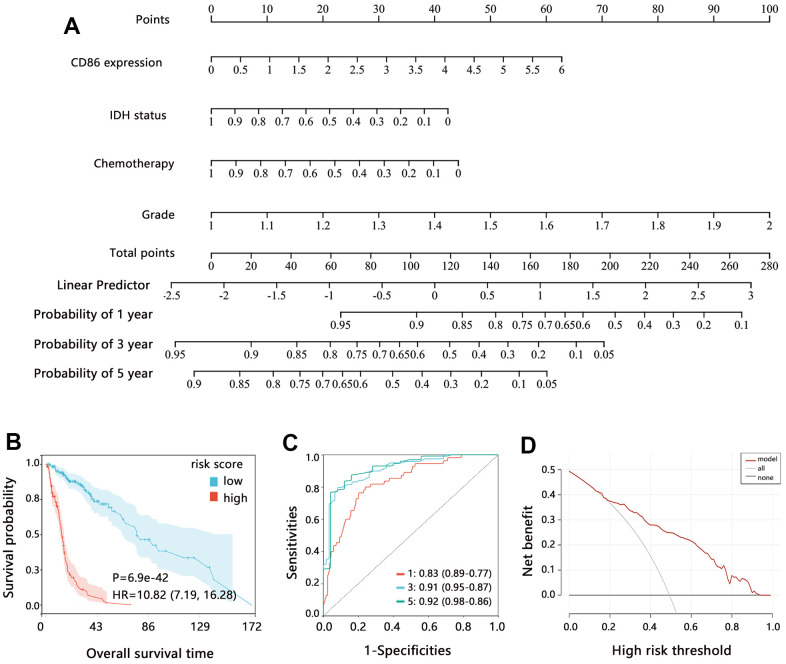
**Assessment of comprehensive nomogram model.** (**A**) Establishment of nomogram model. (**B**) Kaplan-Meier analysis, (**C**) ROC analysis; and (**D**) DCA analysis on the risk score from the nomogram model. Abbreviation: HR, Hazard ratio; CI, confidence interval.

Based on the multivariable Cox regression analysis, we established a comprehensive nomogram model containing the independent factors associated with the OS of patients. The nomogram analysis showed that the comprehensive model had 0.1-0.95, 0.05-0.95, and 0.05-0.9 probabilities for predicting the 1, 3, and 5-year survival, respectively (Figure 4A). Overall C-index value of nomogram model was 0.819 (95%CI: 0.782-0.855, P<0.001). The risk score of the nomogram model was significantly related to the OS, and patients with higher risk scores had poorer prognoses (Figure 4B, HR=10.82, P<0.001). The ROC and DCA analyses showed that risk score had favorable prediction performance (Figure 4C, 4D).

### Potential mechanism exploration of CD86

**Figure 5:**
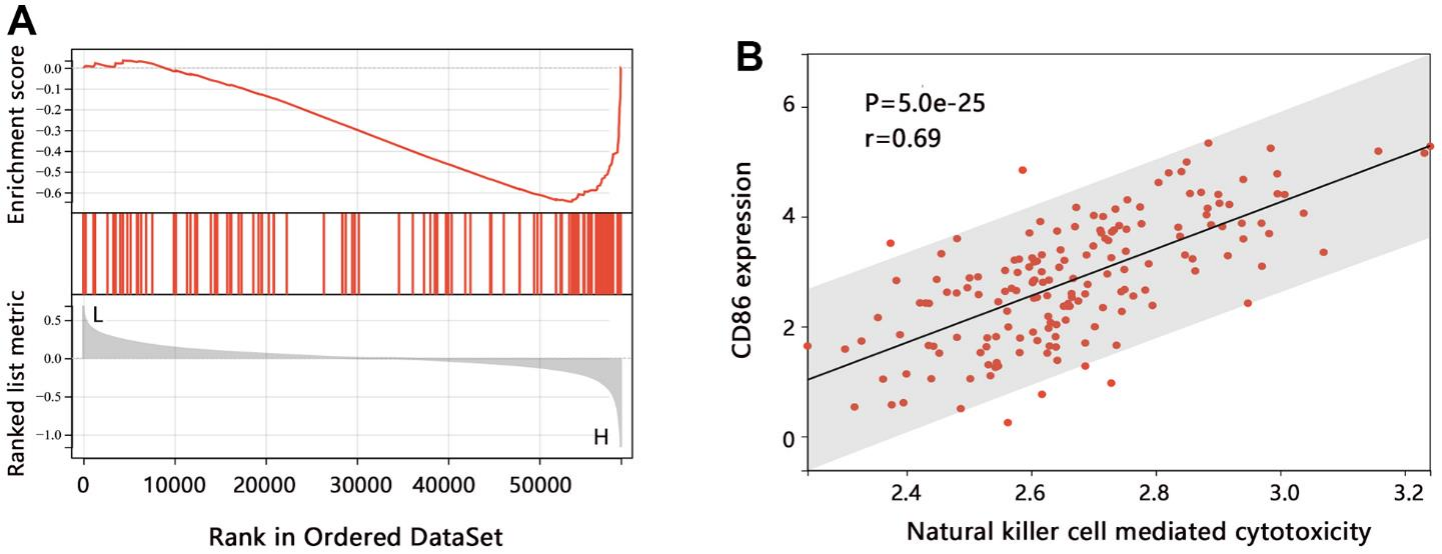
**The pathway enrichment analysis in HGG.** (**A**) GSEA analysis on CD86. (**B**) Relation between the ssGSEA score of each sample and CD86 expression level.

The above analyses have disclosed the potential of CD86 as an important biomarker in HGG, and we then predicted the possible regulatory mechanism associated with CD86. The GSEA enrichment analysis indicated that CD86 was significantly related to the pathway of natural killer (NK) cell-mediated cytotoxicity (Figure 5A, P<0.001), and this way was enriched in the high expression class. We then performed the single sample GSEA and obtained the score of the natural killer cell-mediated cytotoxicity pathway in each sample, finding that the pathway score was positively associated with CD86 expression in HGG patients (Figure 5B, P<0.05). The enrichment analysis showed that CD86 might be related to immunity.

**Figure 6:**
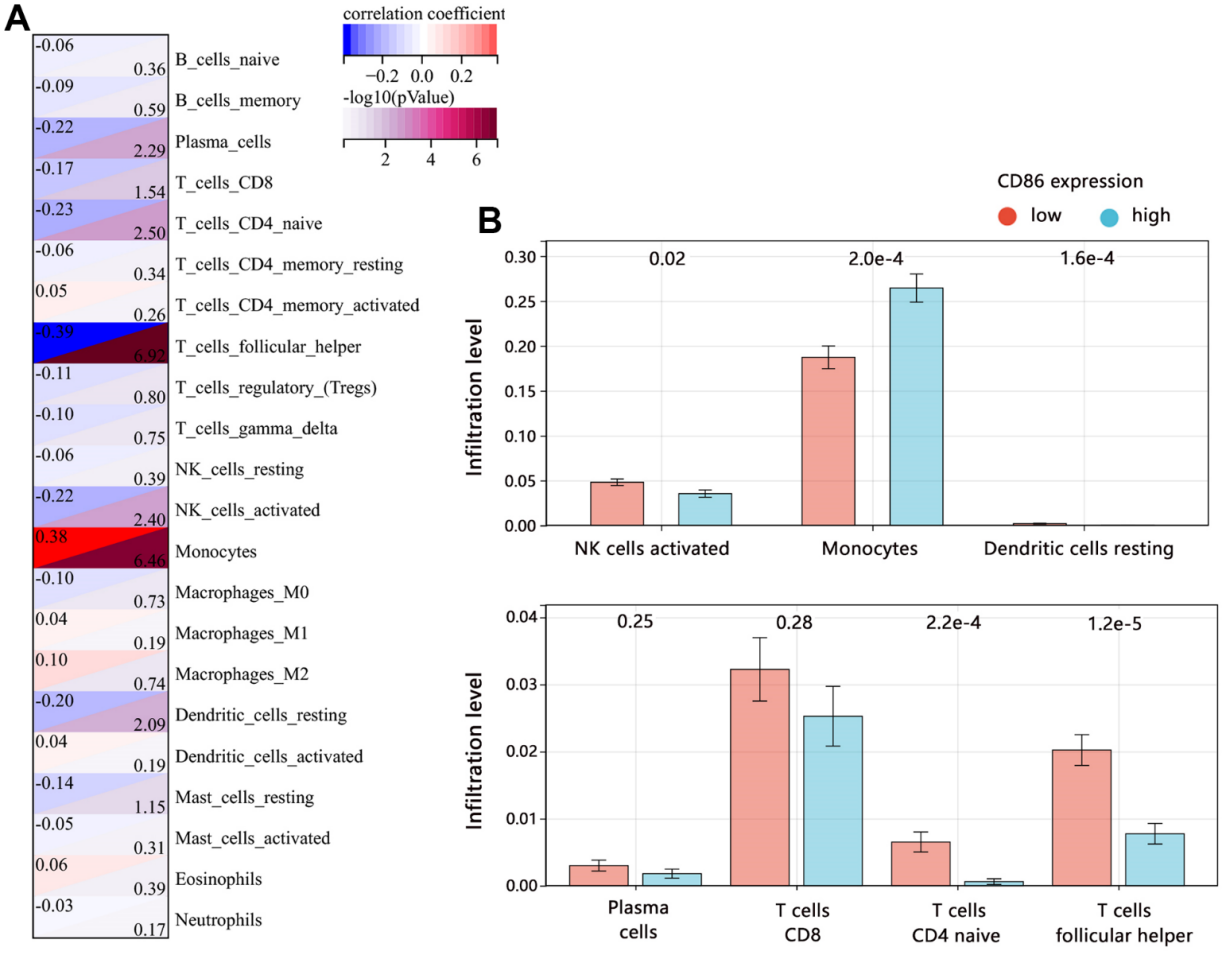
**Correlation between CD86 and immune infiltrates in HGG.** (**A**) Immune microenvironment score by CIBERSORT algorithm. (**B**) The difference in immune infiltration level between CD86 high and low expression groups.

To explore the indicative roles of CD86 on the tumor microenvironment (TME), CIBERSORT was adopted to evaluate the relative proportion of 22 kinds of immune cells. The analysis indicated that CD86 expression showed a negative correlation with the infiltration level of Plasma_cells, T_cells_CD8, T_cells_CD4_naïve, T_cells_follicular_helper, NK_cells_activated, and Dendritic_cells_resting, but positive correlation with Monocytes infiltration (Figure 6A, all P<0.05). Further comparison analysis (Figure 6B) found that the CD86 high expression group had higher infiltration of Monocytes (P<0.001), but lower infiltration of the antitumor immune cell of NK_cells_activated (P=0.02). It followed that CD86 might be significantly involved in the tumor immune microenvironment in HGG.

### Radiomics features extraction and prediction model establishment

The above results have disclosed the potential of CD86 as an immune and prognostic biomarker in HGG. Detection and prediction of CD86 can help us to monitor disease progression and evaluate the immune response. However, the detection of CD86 was an invasive. Therefore, the innovative point of this study is the construction of a noninvasive radiomics model for predicting CD86 expression. We used 2 common modeling approaches to evaluate the application of the model with radiomics features.

**Figure 7:**
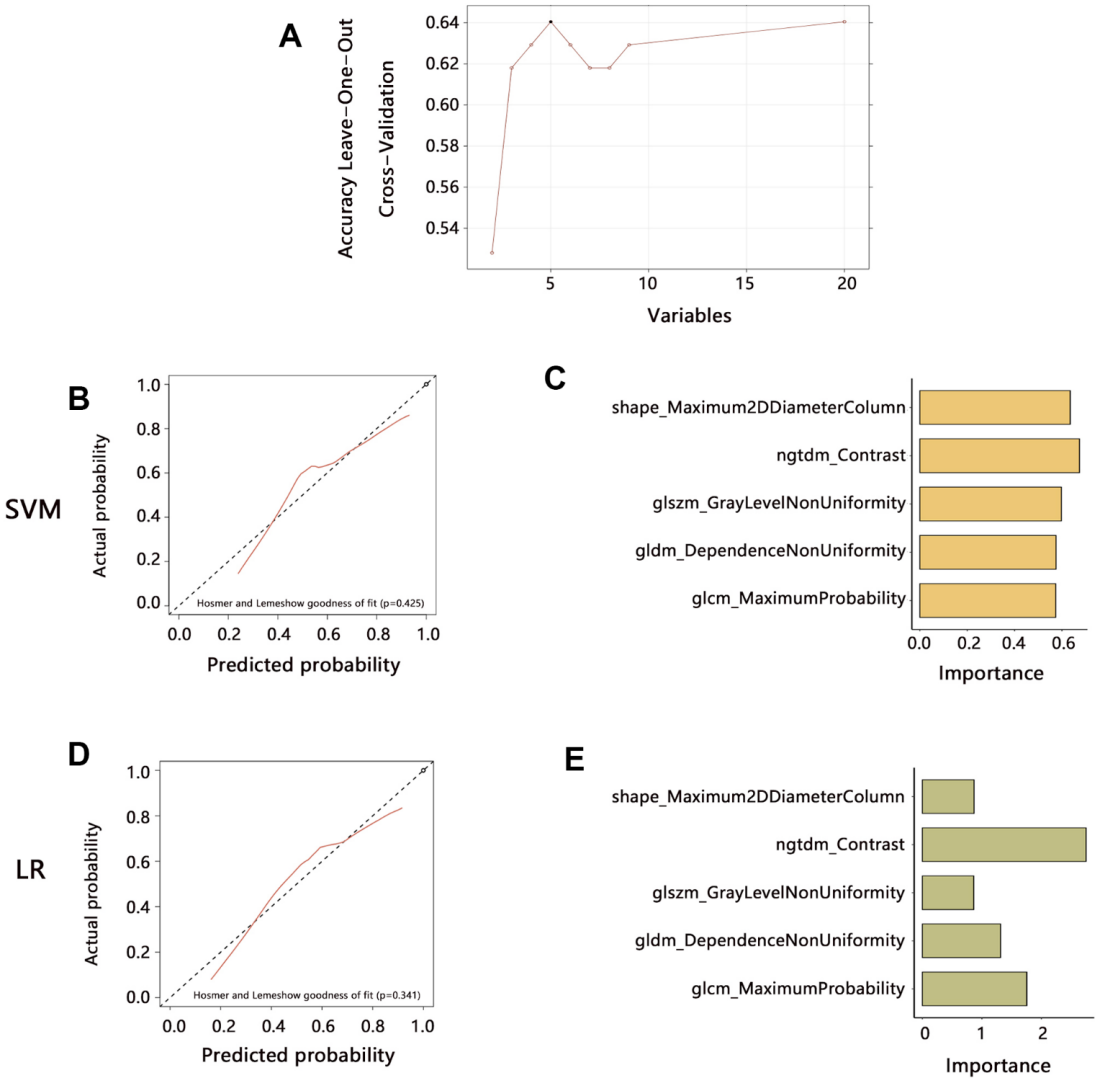
**Radiomics features extraction and prediction model establishment in the training set.** (**A**) The 5 features were finally identified by mRMR and RFE algorithms. (**B**) The prediction performance of the model established by SVM method. (**C**) Variables importance assessment in SVM model. (**D**) The prediction performance of the model established by LR method. (**E**) Variables importance assessment in SVM model. Abbreviation: SVM, Support Vector Machine; LR, Logistic regression.

A total of 89 HGG patients with MRI images and 107 radiomics features were enrolled in the analyses. The patients were first divided into a training set and a validation set according to the 6:4 ratio. Then we developed a model based on the radiomics features for predicting the CD86 expression in the training set. To filter effective features among all the radiomics features, the mRMR algorithm was initially performed and 20 radiomics features were identified. Following by RFE algorithm, 5 most important features were finally determined, including original ngtdm Contrast, original glcm Maximum Probability, original glszm GrayLevelNonUniformity, original gldm DependenceNonUniformity, original shape Maximum2DDiameterColumn (Figure 7A). Further, we established 2 radiomics models with 5 features for predicting CD86 expression by common SVM and LR methods, respectively. The Calibration curves showed that the predicted probabilities of 2 models have favorable consistency with actual probabilities (Figure 7B, 7D). The importance of 5 variables in 2 models was assessed and presented in Figure 7C, 7E.

**Figure 8:**
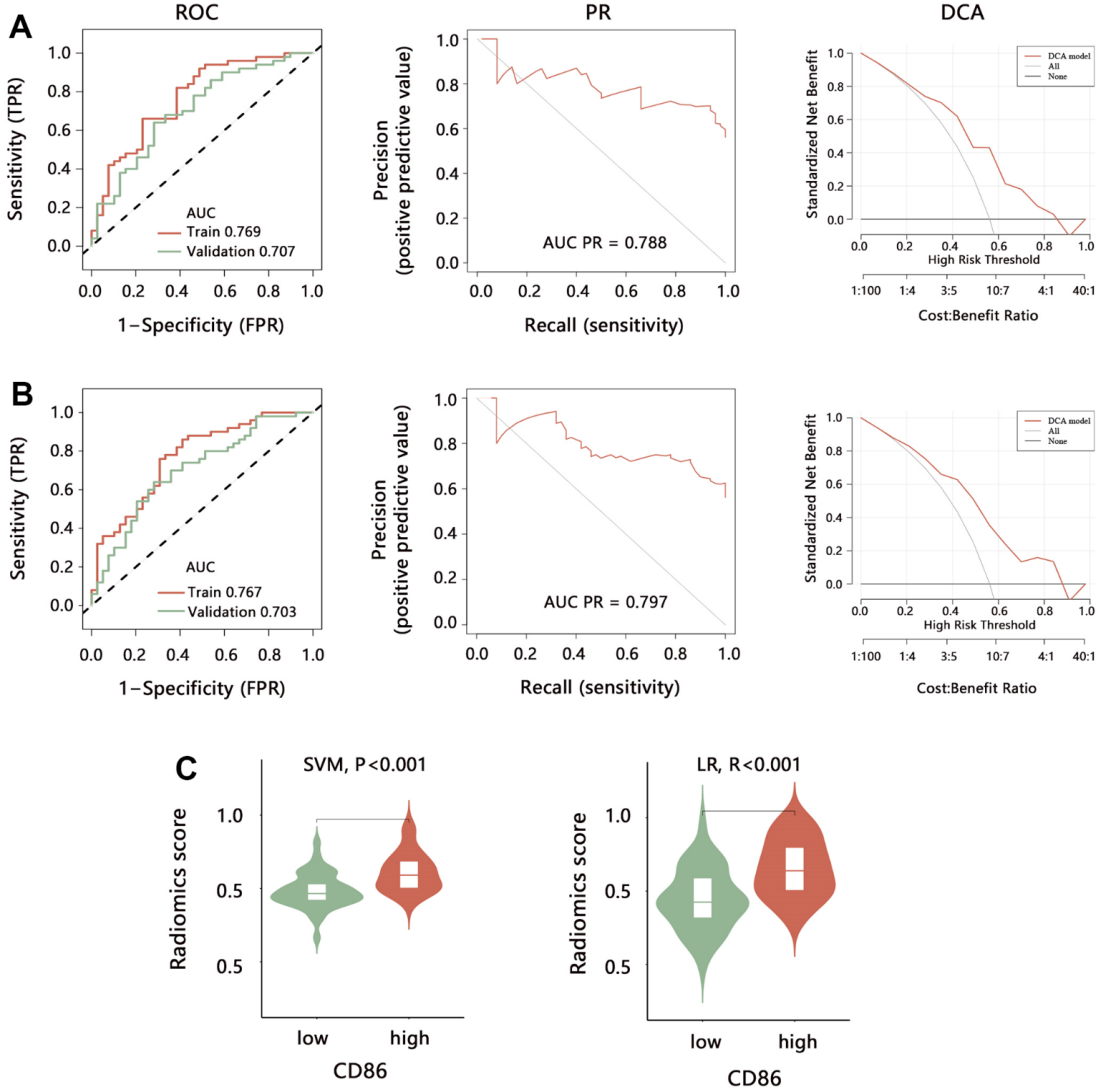
**Performance evaluation of radiomics model for predicting the CD86 expression.** The ROC, PR, and DCA analyses were performed to evaluate the model performance established by (**A**) SVM and (**B**) LR method in training set and validation set. (**C**) The difference in radiomics score between CD86 high and low expression groups. Abbreviation: SVM, Support Vector Machine; LR, Logistic regression; ROC, receiver operating characteristic curve; PR, precision-recall curve; DCA, decision curve analysis; AUC, area under curve.

Further, this study evaluated the prediction performance of 2 models in the training set. ROC analysis (Figure 8A, 8B) indicated that the SVM model has the same prediction ability as the LR model both in training (AUC: 0.769 vs 0.767) and validation sets (AUC: 0.707 vs 0.703). PR curve showed that AUC among the 2 models was similar (0.788 vs 0.797). DCA curve showed that both 2 models achieved more clinical net benefit. We also performed the ROC, PR, and DCA to evaluate the model’s stability for predicting the CD86 expression in the validation set (Figure 8A, 8B), finding that the performance of established models by SVM and LR in the validation set was similar with that in the training set. These results indicated the superiority and stable performance of the radiomics model established with 5 features to predict the CD86 expression. Further, we calculated the radiomics score (RS) according to 5 features in each patient, finding that RS in the CD86 high expression group was significantly higher than that in the low expression group in the 2 models (Figure 8C, all P<0.001).

**Figure 9:**
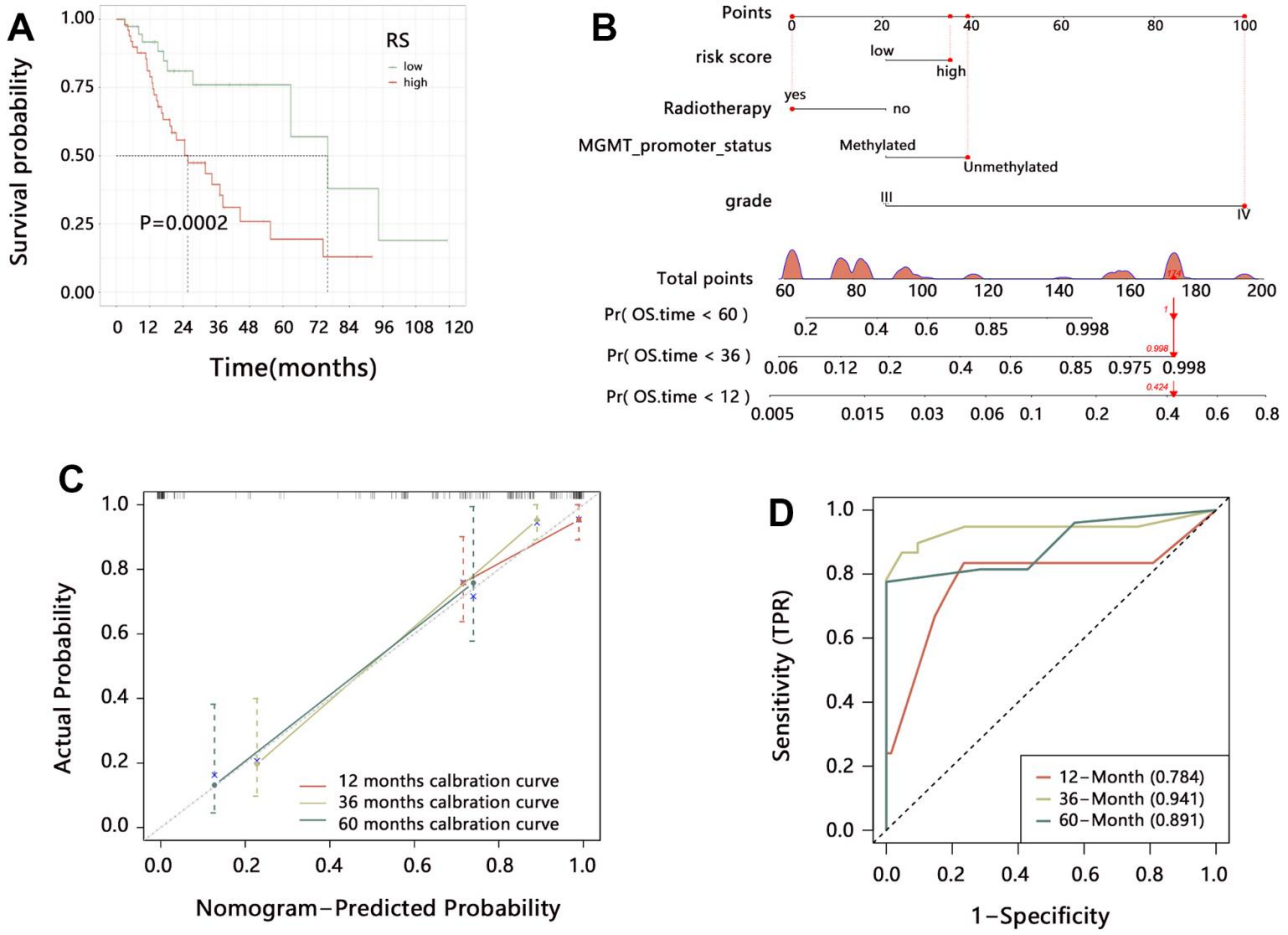
**The clinical value of the radiomics model in HGG.** (**A**) Kaplan-Meier analysis on radiomics score. (**B**) Establishment of comprehensive nomogram model. (**C**) Evaluation of nomogram model with calibration curves. (**D**) Performance assessment of nomogram model with time-dependent ROC analysis.

To further explore the clinical value of the radiomics model in HGG, we analyzed the association of RS with prognosis in HGG. Due to the performance similarity of SVM and LR, we just only evaluate the clinical value of SVM model. Kapan-Meier analysis indicated that a higher RS was related to the shorter OS time (Figure 9A, P<0.001). We then integrated the RS and significant independent prognostic factors, and constructed a comprehensive nomogram model (Figure 9B), finding that the comprehensive model has good predicted probability (Figure 9C). ROC analysis indicated that the nomogram model can favorably predict the 1, 3, and 5-year survival of HGG patients (Figure 9D).

## DISCUSSION

The importance of CD86 as a biomarker in human cancers has been disclosed in previous studies. However, its role in HGG was rarely reported. The present study suggested that CD86 was highly expressed in HGG and it was negatively correlated with OS among HGG patients. We also revealed the potential molecular mechanism and immune microenvironment associated with CD86. Our study initially disclosed the significant clinical value of CD86 in HGG.

Several studies have revealed the role of CD86 among glioma patients. Qiu et al. [12] found that higher expression of CD86 was an independent prognostic indicator for the unfavorable OS of LGG patients by analyzing TCGA, CGGA, as well as their local datasets. Besides, Ahmed et al. [17] suggested that higher CD86 expression was an independent prognostic factor for shorter progression-free survival among newly diagnosed GBM using the TCGA dataset, although it was not significantly correlated with OS. Parney et al. found that CD86/granulocyte-macrophage colony-stimulating factor-transduced glioma-derived cells combined with wild type produced increased cytotoxicity of peripheral blood mononuclear cells, which may help determine an optimal glioma immunogene therapy strategy [18]. Cai et al. found that CD86 was significantly higher in the high glioma-associated stromal cell (GASC) group and showed a positive correlation with the GASC score in all glioma populations and the HGG population [19]. The study also suggested that CD86 may stimulate glioma malignancy. In our research, we found that CD86 was upregulated in HGG compared with the normal group, further, its expression was higher in grade IV than in grade III patients. These results implied that the increase of CD86 expression was positively related to the disease aggravation, and CD86 may be regarded as a monitoring factor in HGG progression. In addition, higher CD86 expression was related to shorter OS time in overall HGG or subgroup patients, and it can independently predict the prognosis of HGG patients. With the increase in the CD86 expression level, the death risk of HGG patients increased. These researches indicated that CD86 might be a promising biomarker in HGG. Monitoring CD86 can help us to evaluate the disease progression and stratify the risk populations with poor prognosis, which contributes to providing individualized treatment plans for patients and improving their quality of life.

CD86, as the ligand for CTLA4 at the cell surface of T cells, could inhibit the activity of T cells by engagement with CTLA4 [20], and similarly, CTLA4 can also capture CD86 [21]. Therefore, CD86 level was significantly associated with the activity of T cells and the anti-CTLA4 immunotherapy response. Immunotherapy has become a promising strategy for the treatment of cancers, and it can penetrate the blood-brain barrier [22]. However, these clinical trials have indicated limited efficacy in glioma. A recent study provided a novel synergic immunotherapeutic strategy that combined immune checkpoint blockade treatment with ferroptosis inhibition in glioma [23]. Therefore, CD86 and ferroptosis inhibition may be a potentially useful strategy in the treatment of HGG, which can be regarded as new research direction associated with CD86 in HGG. Our study also found that CD86 was significantly related to the NK cell-mediated cytotoxicity. Wu et al. found a strong association between NK cell abundances and immune checkpoint proteins of CD86 in hepatocellular carcinoma [24]. Lichtenegger et al. found that CD86 was a key player for T helper 1 polarization and NK cell activation by Toll-like receptor-induced dendritic cells [25]. Harnack et al. found that the human NK-like cell line YT could be useful in the immunotherapy of patients with CD86 (+) multiple myeloma [26]. Peng et al. found that ligation of CD86 with CTLA4Ig significantly increased the ability of NK cells to kill tumor cells [27]. These researches indicated that the expression of CD86 was significantly related to the activity of NK and T cells, and might influence the response of immune therapy.

Our study has shown that CD86 is a potential prognosis and immunotherapy biomarker in HGG. Detection and prediction of CD86 can stratify the high-risk populations with HGG, and contribute to evaluating immunotherapy response. However, the detection of CD86 was invasive. Radiomics has become a promising and non-invasive method for the prediction of genotyping and the expression of specific molecules for gliomas. Choi et al. demonstrated that the hybrid radiomics model could predict the IDH status of gliomas, showing highly reproducible and generalizable [28]. Li et al. reported that multiparameter MRI-based radiomics signature could predict the MGMT promoter methylation in glioblastoma multiforme and astrocytoma, respectively [29]. Tian et al. suggested that a multiparametric MRI-based radiomics model could predict the TERT promoter mutations in HGG [30]. Chen et al. indicated multiparametric MRI-based radiomics model could predict the PTEN mutation status in patients with glioma [31]. Wang et al. suggested that T2-FLAIR (Fluid-attenuated inversion recovery) based radiomics features could effectively predict the expression levels of CD44 and CD133 for LGG patients [32]. The models based on radiomics features for predicting molecular expression have been widely applied.

Our study also constructed a 5-features signature for predicting CD86 expression, and the results showed that the signature had stable and favorable performance. Besides the clinical value in predicting the molecular marker, the radiomics model also presented the potential to predict the patient’s prognosis. Yan et al. found that MRI-based radiomics may be useful for noninvasively predicting progression-free survival and OS in gliomas regardless of grades with a C-index of 0.736 and 0.735, respectively [33]. Zhang et al. found that the radiomics model performed better than clinical risk factors in the survival stratification of patients with GBM, and it exhibited more promising discrimination when the radiomics model and clinical risk factors were combined [34]. Our study also found that the radiomics model could favorably distinguish the prognosis of HGG patients. The prediction performance was better after combining the radiomics model and clinical factors.

The present study also has some limitations. First, this is a retrospective study with a limited sample size and a lack of external data validation. The stability and generalizability of the established models cannot be verified. In addition, although there were several studies reported the value of the radiomics model in predicting molecular biomarkers, no research reported the appilicaiton of radiomics model in predicting CD86. Therefore, we failed to compare the performance of our radiomics model with others. Second, only the mRNA expression of CD86 was investigated, while its protein expression was not detected because of some objective reasons such as a lack of samples. Besides, only the CE-T1WI images were analyzed for radiomics analysis, which could limit the feasibility of the model. However, to our knowledge, this is the first study to explore the clinical value of CD86 in HGG, and we also innovatively predict the CD86 by a non-invasive radiomics model, which might provide valuable references in this field.

## CONCLUSIONS

This study found that CD86 was abnormally expressed in HGG compared with that in the control group. Higher expression was observed in patients with age≥60 years, non-codel of Chr_1p_19q, unmethylated MGMT promoter, wildtype IDH, and grade IV. We also disclosed the significant correlation of CD86 higher expression with the poor prognosis of patients, and CD86 was proved to be an independent prognostic factor in HGG. After integrating the CD86 with clinical characteristics, the probability of a patient’s survival can be predicted favorably. Our study initially confirmed the potential of CD86 as a promising molecular biomarker in HGG. Pathway analysis indicated that CD86 was related to NK cell-mediated cytotoxicity. Further, we established a radiomics model based on MRI images to non-invasively predict the CD86 expression, finding that the radiomics model had stable prediction performance on CD86. Our study provided a potential therapeutic target for HGG and established a non-invasive prediction model, which may contribute to the prognosis improvement of HGG and control of disease progression.

## References

[1] Liu A, Zhao H, Sun B, Han X, Zhou D, Cui Z, Ma X, Zhang J, Yuan L. A predictive analysis approach for paediatric and adult high-grade glioma: miRNAs and network insight. Ann Transl Med. 2020; 8:242. 10.21037/atm.2020.01.12 32309389 PMC7154480

[2] Marinari E, Allard M, Gustave R, Widmer V, Philippin G, Merkler D, Tsantoulis P, Dutoit V, Dietrich PY. Inflammation and lymphocyte infiltration are associated with shorter survival in patients with high-grade glioma. Oncoimmunology. 2020; 9:1779990. 10.1080/2162402X.2020.1779990 32923142 PMC7458651

[3] Wu XF, Liang X, Wang XC, Qin JB, Zhang L, Tan Y, Zhang H. Differentiating high-grade glioma recurrence from pseudoprogression: Comparing diffusion kurtosis imaging and diffusion tensor imaging. Eur J Radiol. 2021; 135:109445. 10.1016/j.ejrad.2020.109445 33341429

[4] Detti B, Scoccianti S, Teriaca MA, Maragna V, Lorenzetti V, Lucidi S, Bellini C, Greto D, Desideri I, Livi L. Bevacizumab in recurrent high-grade glioma: a single institution retrospective analysis on 92 patients. Radiol Med. 2021; 126:1249–54. 10.1007/s11547-021-01381-5 34081269 PMC8370943

[5] Halliday N, Williams C, Kennedy A, Waters E, Pesenacker AM, Soskic B, Hinze C, Hou TZ, Rowshanravan B, Janman D, Walker LS, Sansom DM. CD86 Is a Selective CD28 Ligand Supporting FoxP3+ Regulatory T Cell Homeostasis in the Presence of High Levels of CTLA-4. Front Immunol. 2020; 11:600000. 10.3389/fimmu.2020.600000 33363541 PMC7753196

[6] Liu PC, Ssu CT, Tsao YP, Liou TL, Tsai CY, Chou CT, Chen MH, Leu CM. Cytotoxic T lymphocyte-associated antigen-4-Ig (CTLA-4-Ig) suppresses Staphylococcus aureus-induced CD80, CD86, and pro-inflammatory cytokine expression in human B cells. Arthritis Res Ther. 2020; 22:64. 10.1186/s13075-020-2138-x 32228715 PMC7106629

[7] Kennedy A, Waters E, Rowshanravan B, Hinze C, Williams C, Janman D, Fox TA, Booth C, Pesenacker AM, Halliday N, Soskic B, Kaur S, Qureshi OS, et al. Differences in CD80 and CD86 transendocytosis reveal CD86 as a key target for CTLA-4 immune regulation. Nat Immunol. 2022; 23:1365–78. 10.1038/s41590-022-01289-w 35999394 PMC9477731

[8] de Vos L, Grünwald I, Bawden EG, Dietrich J, Scheckenbach K, Wiek C, Zarbl R, Bootz F, Landsberg J, Dietrich D. The landscape of *CD28, CD80, CD86, CTLA4*, and *ICOS* DNA methylation in head and neck squamous cell carcinomas. Epigenetics. 2020; 15:1195–212. 10.1080/15592294.2020.1754675 32281488 PMC7595594

[9] van Gool SW, Barcy S, Devos S, Vandenberghe P, Ceuppens JL, Thielemans K, de Boer M. CD80 (B7-1) and CD86 (B7-2): potential targets for immunotherapy? Res Immunol. 1995; 146:183–96. 10.1016/0923-2494(96)80256-2 8525052

[10] Takasaki Y, Abe K, Tokano Y, Hashimoto H. The expression of LFA-1, ICAM-1, CD80 and CD86 molecules in lupus patients: implication for immunotherapy. Intern Med. 1999; 38:175–7. 10.2169/internalmedicine.38.175 10225679

[11] Geng P, Zhao X, Xiang L, Liao Y, Wang N, Ou J, Xie G, Liu C, Li J, Li H, Zeng R, Liang H. Distinct role of CD86 polymorphisms (rs1129055, rs17281995) in risk of cancer: evidence from a meta-analysis. PLoS One. 2014; 9:e109131. 10.1371/journal.pone.0109131 25369324 PMC4219668

[12] Qiu H, Tian W, He Y, Li J, He C, Li Y, Liu N, Li J. Integrated Analysis Reveals Prognostic Value and Immune Correlates of CD86 Expression in Lower Grade Glioma. Front Oncol. 2021; 11:654350. 10.3389/fonc.2021.654350 33954112 PMC8089378

[13] Bi Y, Wu ZH, Cao F. Prognostic value and immune relevancy of a combined autophagy-, apoptosis- and necrosis-related gene signature in glioblastoma. BMC Cancer. 2022; 22:233. 10.1186/s12885-022-09328-3 35241019 PMC8892733

[14] Li Z, Liu L, Zhang Z, Yang X, Li X, Gao Y, Huang K. A Novel CT-Based Radiomics Features Analysis for Identification and Severity Staging of COPD. Acad Radiol. 2022; 29:663–73. 10.1016/j.acra.2022.01.004 35151548

[15] Yu Q, Wang A, Gu J, Li Q, Ning Y, Peng J, Lv F, Zhang X. Multiphasic CT-Based Radiomics Analysis for the Differentiation of Benign and Malignant Parotid Tumors. Front Oncol. 2022; 12:913898. 10.3389/fonc.2022.913898 35847942 PMC9280642

[16] Zheng Y, Zhou D, Liu H, Wen M. CT-based radiomics analysis of different machine learning models for differentiating benign and malignant parotid tumors. Eur Radiol. 2022; 32:6953–64. 10.1007/s00330-022-08830-3 35484339

[17] Ahmed MH, Hernández-Verdin I, Bielle F, Verreault M, Lerond J, Alentorn A, Sanson M, Idbaih A. Expression and Prognostic Value of CD80 and CD86 in the Tumor Microenvironment of Newly Diagnosed Glioblastoma. Can J Neurol Sci. 2023; 50:234–42. 10.1017/cjn.2022.5 35022089

[18] Parney IF, Farr-Jones MA, Kane K, Chang LJ, Petruk KC. Human autologous *in vitro* models of glioma immunogene therapy using B7-2, GM-CSF, and IL12. Can J Neurol Sci. 2002; 29:267–75. 10.1017/s0317167100002055 12195617

[19] Cai X, Yuan F, Zhu J, Yang J, Tang C, Cong Z, Ma C. Glioma-Associated Stromal Cells Stimulate Glioma Malignancy by Regulating the Tumor Immune Microenvironment. Front Oncol. 2021; 11:672928. 10.3389/fonc.2021.672928 33996602 PMC8117153

[20] Naimi A, Mohammed RN, Raji A, Chupradit S, Yumashev AV, Suksatan W, Shalaby MN, Thangavelu L, Kamrava S, Shomali N, Sohrabi AD, Adili A, Noroozi-Aghideh A, Razeghian E. Tumor immunotherapies by immune checkpoint inhibitors (ICIs); the pros and cons. Cell Commun Signal. 2022; 20:44. 10.1186/s12964-022-00854-y 35392976 PMC8991803

[21] Wong C, Darby JM, Murphy PR, Pinfold TL, Lennard PR, Woods GM, Lyons AB, Flies AS. Tasmanian devil CD28 and CTLA4 capture CD80 and CD86 from adjacent cells. Dev Comp Immunol. 2021; 115:103882. 10.1016/j.dci.2020.103882 33039410

[22] Xu S, Tang L, Li X, Fan F, Liu Z. Immunotherapy for glioma: Current management and future application. Cancer Lett. 2020; 476:1–12. 10.1016/j.canlet.2020.02.002 32044356

[23] Liu T, Zhu C, Chen X, Guan G, Zou C, Shen S, Wu J, Wang Y, Lin Z, Chen L, Cheng P, Cheng W, Wu A. Ferroptosis, as the most enriched programmed cell death process in glioma, induces immunosuppression and immunotherapy resistance. Neuro Oncol. 2022; 24:1113–25. 10.1093/neuonc/noac033 35148413 PMC9248406

[24] Wu M, Mei F, Liu W, Jiang J. Comprehensive characterization of tumor infiltrating natural killer cells and clinical significance in hepatocellular carcinoma based on gene expression profiles. Biomed Pharmacother. 2020; 121:109637. 10.1016/j.biopha.2019.109637 31810126

[25] Lichtenegger FS, Mueller K, Otte B, Beck B, Hiddemann W, Schendel DJ, Subklewe M. CD86 and IL-12p70 are key players for T helper 1 polarization and natural killer cell activation by Toll-like receptor-induced dendritic cells. PLoS One. 2012; 7:e44266. 10.1371/journal.pone.0044266 22962607 PMC3433478

[26] Harnack U, Johnen H, Pecher G. Natural killer cell line YT exerts cytotoxicity against CD86+ myeloma cells. Anticancer Res. 2011; 31:475–9. 21378326

[27] Peng Y, Luo G, Zhou J, Wang X, Hu J, Cui Y, Li XC, Tan J, Yang S, Zhan R, Yang J, He W, Wu J. CD86 is an activation receptor for NK cell cytotoxicity against tumor cells. PLoS One. 2013; 8:e83913. 10.1371/journal.pone.0083913 24349559 PMC3859666

[28] Choi YS, Bae S, Chang JH, Kang SG, Kim SH, Kim J, Rim TH, Choi SH, Jain R, Lee SK. Fully automated hybrid approach to predict the IDH mutation status of gliomas via deep learning and radiomics. Neuro Oncol. 2021; 23:304–13. 10.1093/neuonc/noaa177 32706862 PMC7906063

[29] He J, Ren J, Niu G, Liu A, Wu Q, Xie S, Ma X, Li B, Wang P, Shen J, Wu J, Gao Y. Multiparametric MR radiomics in brain glioma: models comparation to predict biomarker status. BMC Med Imaging. 2022; 22:137. 10.1186/s12880-022-00865-8 35931979 PMC9354364

[30] Tian H, Wu H, Wu G, Xu G. Noninvasive Prediction of TERT Promoter Mutations in High-Grade Glioma by Radiomics Analysis Based on Multiparameter MRI. Biomed Res Int. 2020; 2020:3872314. 10.1155/2020/3872314 32509858 PMC7245686

[31] Chen H, Lin F, Zhang J, Lv X, Zhou J, Li ZC, Chen Y. Deep Learning Radiomics to Predict PTEN Mutation Status From Magnetic Resonance Imaging in Patients With Glioma. Front Oncol. 2021; 11:734433. 10.3389/fonc.2021.734433 34671557 PMC8521070

[32] Wang Z, Tang X, Wu J, Zhang Z, He K, Wu D, Chen S, Xiao X. Radiomics features based on T2-weighted fluid-attenuated inversion recovery MRI predict the expression levels of *CD44* and *CD133* in lower grade gliomas. Future Oncol. 2022; 18:807–19. 10.2217/fon-2021-1173 34783576

[33] Yan J, Zhang B, Zhang S, Cheng J, Liu X, Wang W, Dong Y, Zhang L, Mo X, Chen Q, Fang J, Wang F, Tian J, et al. Quantitative MRI-based radiomics for noninvasively predicting molecular subtypes and survival in glioma patients. NPJ Precis Oncol. 2021; 5:72. 10.1038/s41698-021-00205-z 34312469 PMC8313682

[34] Zhang X, Lu H, Tian Q, Feng N, Yin L, Xu X, Du P, Liu Y. A radiomics nomogram based on multiparametric MRI might stratify glioblastoma patients according to survival. Eur Radiol. 2019; 29:5528–38. 10.1007/s00330-019-06069-z 30847586

